# Impact of Serum γ-Glutamyltransferase on Overall Survival in Men with Metastatic Castration-Resistant Prostate Cancer Treated with Docetaxel

**DOI:** 10.3390/cancers13215587

**Published:** 2021-11-08

**Authors:** Minami Une, Kosuke Takemura, Kentaro Inamura, Hiroshi Fukushima, Masaya Ito, Shuichiro Kobayashi, Takeshi Yuasa, Junji Yonese, Philip G. Board, Fumitaka Koga

**Affiliations:** 1 Department of Urology, Tokyo Metropolitan Cancer and Infectious Diseases Center Komagome Hospital, Tokyo 113-8677, Japan; 1203373miu@cick.jp (M.U.); ito.uro@cick.jp (M.I.); shu_kobayashi@cick.jp (S.K.); f-koga@cick.jp (F.K.); 2 Department of Urology, Cancer Institute Hospital of Japanese Foundation for Cancer Research, Tokyo 135-8550, Japan; takeshi.yuasa@jfcr.or.jp (T.Y.); jyonese@jfcr.or.jp (J.Y.); 3 Department of Pathology, Cancer Institute Hospital of Japanese Foundation for Cancer Research, Tokyo 135-8550, Japan; kentaro.inamura@jfcr.or.jp; 4 Department of Urology, Tokyo Medical and Dental University, Tokyo 113-8519, Japan; fukuuro@tmd.ac.jp; 5 ACRF Department of Cancer Biology and Therapeutics, Molecular Genetics Group, John Curtin School of Medical Research, Australian National University, Canberra, ACT 2601, Australia; Philip.G.Board@anu.edu.au

**Keywords:** γ-glutamyltransferase, prognosis, prostatic neoplasms, serologic tests

## Abstract

**Simple Summary:**

γ-Glutamyltransferase (GGT) is a biomarker of oxidative stress and its elevation in the serum is linked to poor survival in various malignancies; however, reports on metastatic castration-resistant prostate cancer (mCRPC) are scarce. Moreover, the source of serum GGT in men with mCRPC is largely unknown. The aims of this study were to determine the impact of serum GGT on overall survival in men with mCRPC receiving docetaxel therapy, and to examine the association between systemic and local GGT levels using immunohistochemistry. Of note, high serum GGT was associated with adverse overall survival as were low hemoglobin and high prostate-specific antigen levels. Additionally, tissue GGT expression status in prostate specimens was moderately positively associated with serum GGT. We demonstrated that pre-therapeutic serum GGT was an independent prognosticator in men with mCRPC receiving docetaxel therapy, and that overexpression of GGT in cancer cells might be responsible for the elevation of serum GGT.

**Abstract:**

Background: Reports on the prognostic significance of serum γ-glutamyltransferase (GGT) in men with metastatic castration-resistant prostate cancer (mCRPC) are limited. In addition, GGT expression status in cancer tissues has not been well characterized regardless of cancer types. Methods: This retrospective study included 107 consecutive men with mCRPC receiving docetaxel therapy. The primary endpoints were associations of serum GGT with overall survival (OS) and prostate-specific antigen (PSA) response. The secondary endpoint was an association of serum GGT with progression-free survival (PFS). Additionally, GGT expression status was immunohistochemically semi-quantified using tissue microarrays. Results: A total of 67 (63%) men died during follow-up periods (median 22.5 months for survivors). On multivariable analysis, high Log GGT was independently associated with adverse OS (HR 1.49, *p* = 0.006) as were low hemoglobin (HR 0.79, *p* = 0.002) and high PSA (HR 1.40, *p* < 0.001). In contrast, serum GGT was not significantly associated with PSA response or PFS. Moreover, incorporation of serum GGT into established prognostic models (i.e., Halabi and Smaletz models) increased their C-indices for predicting OS from 0.772 to 0.787 (*p* = 0.066) and from 0.777 to 0.785 (*p* = 0.118), respectively. Furthermore, there was a positive correlation between serum and tissue GGT levels (ρ = 0.53, *p* = 0.003). Conclusions: Serum GGT may be a prognostic biomarker in men with mCRPC receiving docetaxel therapy. GGT overexpression by prostate cancer cells appears to be responsible for the elevation of GGT in the serum.

## 1. Introduction

Prostate cancer (PC) is one the most commonly occurring neoplasms in men, with 1.6 million newly diagnosed each year, and 366,000 deaths caused by the disease [1]. Prognosis of men with metastatic castration-resistant PC (mCRPC) is especially poor, even with docetaxel therapy, giving a median overall survival (OS) of approximately 17 months with docetaxel according to the TRAPEZE randomized clinical trial [2]. In the sequential treatment of mCRPC, combined androgen blockade therapies including both gonadotropin-releasing hormone analogues and androgen-directed strategies have been considered a cornerstone [3]. Additionally, docetaxel treatment has been the standard treatment for mCRPC as it can extend median OS by 2–3 months on the basis of findings in TAX-327 and SWOG 9916 trials [4,5]. Docetaxel is therefore recommended for both symptomatic and asymptomatic men with mCRPC even though special caution is required for those with poor performance status (PS) due to symptoms directly associated with high tumor burden [6]. Thus far, there are several established nomograms for predicting OS in men with mCRPC. Halabi et al. presented a nomogram consisting of visceral disease, Gleason sum, PS, hemoglobin, lactate dehydrogenase (LDH), alkaline phosphatase (ALP), and prostate-specific antigen (PSA) [7]. They later updated the model by adding opioid use and albumin to their original model [8]. Smaletz et al. also presented a prognostic model comprising age, PS, hemoglobin, albumin, LDH, ALP, and PSA [9]. Previously, C-reactive protein (CRP), a biomarker reflecting systemic inflammatory response, was also reported to be an independent prognostic factor in men with mCRPC receiving docetaxel therapy [10].

γ-Glutamyltransferase (GGT) contributes to the metabolism of glutathione (GSH), a main intracellular antioxidant, and is widely distributed in the human body especially strongly in the kidney and liver, while the luminal surface of the normal prostatic glandular epithelium is GGT-positive, in contrast to underlying basal epithelial cells that are GGT-negative [11]. The extracellular domain of GGT degrades the glutamyl bond of GSH and releases cysteinyl–glycine and a glutamyl amino acid, which is the first step of the glutamyl cycle [12]. In cancer cells, overexpressed GGT promotes proliferation by recycling cysteine from the extracellular GSH, which increases resistance to oxidative stress [13]. A prospective cohort of a middle-aged, male population demonstrated that serum GGT was positively and independently associated with future risk of PC over long-term follow-up [14]. A recent systematic review suggested that GGT could be detected in the peripheral blood of patients with advanced genitourinary cancer particularly when cancer cells had high GGT expression, the disease was advanced, or the tumor burden was heavy [15]. Although the source of serum GGT in men with mCRPC is largely unknown, it could be derived from PC cells, given that higher immunopositivity of GGT was observed in prostatic adenocarcinomas than in normal prostatic cells based on previous immunohistochemical analysis [16], and that cultured PC cells released soluble GGT complexes into supernatants [17]. Several reports having suggested a joint effect between serum and tissue GGT levels [18,19,20], no study to date has ever compared systemic and local GGT levels in men with mCRPC. In addition, a previous study showed that transfection of GGT into cultured PC3 cells provided the cells with high levels of cysteine, making GGT-positive cells more resistant to the toxicity of cisplatin [21]. An aggressive phenotype of GGT-positive PC cells could further be supported by the evidence that transcription of GGT was activated by tumor necrosis factor-α and nuclear factor-κB [22], both of which are known to promote the biological aggressiveness of PC cells [23].

Based on the previous findings discussed above, we hypothesized that pre-therapeutic serum GGT would reflect an aggressive phenotype of mCRPC and that serum GGT may be applied as a prognosticator as well as predictor for PSA response and progression-free survival (PFS) in men with mCRPC receiving docetaxel therapy. We also immunohistochemically investigated tissue GGT expression status so that they could be compared with serum GGT in men with mCRPC. The aims of this study are, therefore, to investigate the prognostic significance of serum GGT in men with mCRPC receiving docetaxel therapy, and to compare the systemic and local GGT levels using tissue microarrays.

## 2. Materials and Methods

### 2.1. Study Design

A total of 114 consecutive men with mCRPC receiving docetaxel therapy at a single tertiary cancer center in Tokyo, Japan between 2008 and 2019 were retrospectively reviewed by two authors (M.U. and K.T.). When an outlier was suspected, an additional reviewer (M.I.) was enrolled and the medical record was re-evaluated for clarification. Of the 114 men, five and two were excluded due to unavailable laboratory data and missing follow-up data, respectively. Accordingly, 107 men were subjects of the present study. Variables collected from medical records included age, Eastern Cooperative Oncology Group (ECOG) PS, Gleason score, metastatic sites, opioid use, pre-docetaxel sequential treatments, time from diagnosis to docetaxel, cycles of docetaxel, post-docetaxel sequential treatment, and the following data were measured at a clinical laboratory center at docetaxel initiation: hemoglobin, albumin, GGT, LDH, ALP, CRP, and PSA. Cut-off values of ECOG PS ≥ 1, Gleason sum ≥ 8, and LDH > upper limit of normal (ULN) followed those in original publications by Halabi et al. [7,8]. ALP, GGT, and PSA were logarithmically transformed in the following statistical analyses [24]. Docetaxel was administered at a dose of 60 mg/m^2^ every three weeks to maintain similar efficacy with an acceptable toxicity profile compared to the standard 75 mg/m^2^ regimen as reported among Japanese men [25].

The primary endpoints were OS and PSA response, whereas the secondary endpoint was PFS determined by post-therapeutic PSA and radiographical changes. In the present study, OS was defined as the time from docetaxel initiation to either death or the last follow-up. PSA response was defined as PSA reduction of 50% or greater from baseline at 12 weeks after docetaxel initiation and was therefore analyzed in a subgroup of men with mCRPC who received four or more cycles of docetaxel. PSA progression was defined as a 25% increase and absolute value 2.0 ng/mL increase above the baseline according to the definition by the Prostate Cancer Clinical Trials Working Group 3 [26]. Radiographical progression was defined as a 20% or greater increase in the sum of the longest diameter of measured lesions (target lesions) or two new bone metastatic lesions according to the revised Response Evaluation Criteria in Solid Tumors (RECIST) guideline [27]. PFS was defined as the time from docetaxel initiation to either PSA progression, radiographic progression, or death, whichever occurred first. 

To further test the hypothesis that systemic serum GGT would reflect local GGT expression, serum GGT in 29 men with de novo, metastatic, castration-sensitive PC (mCSPC) was compared with tissue GGT expression status determined by immunohistochemical analysis. The Institutional Review Board approved the present study protocol (approval numbers: 2242 and 2018-1177, data cut-off date: 1 March 2021). 

### 2.2. Immunohistochemistry

Formalin-fixed paraffin-embedded prostate biopsy samples were reassembled into multiple tissue microarrays, which were collected from representative paraffin blocks so that reaction conditions could be normalized. Heat-induced epitope retrieval was performed at 100 °C for 20 min in 1 mmol/L ethylenediaminetetraacetic acid buffer (pH 9.0). Slides were incubated with a primary mouse monoclonal antibody against GGT1 (clone 1F9, dilution 1:800, Abcam, Cambridge, UK) at room temperature for 15 min. The VECTASTAIN Elite ABC HRP Kit (Vector Laboratories, Burlingame, CA, USA) and peroxidase detection methods were used for antibody detection. Tissue GGT expression status was semi-quantified based on the intensity of staining (i.e., negative = 0, weak = 1, moderate = 2, and strong = 3) as described previously [18,20]. Two authors (M.U. and K.T.) evaluated the samples independently. After inter-observer agreement was assessed, discrepancies were resolved by re-evaluation and discussion to reach consensus.

### 2.3. Statistical Analyses

Associations of variables with OS and PFS were analyzed by Cox proportional hazards model. A reduced multivariable model was generated from all variables included in univariable analysis by backward elimination of the variable with the highest *p*-value from each iteration of the multivariable analysis. Harrell’s concordance index (C-index) was used to estimate the predictive accuracy of prognostic models [28]. Logistic regression analysis was carried out to assess clinicopathological parameters for predicting PSA response. A Cohen’s kappa coefficient was calculated to assess the level of inter-observer agreement in immunohistochemical analysis. The degree of correlation between serum and tissue GGT levels was assessed by Spearman’s rank order correlation coefficient. Differences in variables between two or more groups were evaluated by Mann–Whitney U test, Kruskal–Wallis test, or Fisher’s exact test. All statistical analyses were performed with JMP PRO 15.2.0 (SAS Institute Inc., Cary, NC, USA) and R 4.1.0 (R Foundation for Statistical Computing, Vienna, Austria). A two-sided *p*-value < 0.05 was considered statistically significant.

## 3. Results

### 3.1. Characteristics of 107 mCRPC Men Treated with Docetaxel (Full Cohort)

The demographics of 107 men with mCRPC are listed in Table 1. The median (interquartile range [IQR]) age at docetaxel initiation was 72.6 (68.7–76.7) years. ECOG PS was 0 and ≥1 in 84 (79%) and 23 (21%) men, respectively. Gleason sum was <8, ≥8, and unknown in 8 (7%), 89 (83%), and 10 (9%), respectively. Lymph node metastasis only, bone (and lymph node) metastasis, and any visceral metastasis were observed in 7 (7%), 82 (77%), and 18 (17%), respectively. A total of 19 (18%) men were opioid users. All men (*n* = 107, 100%) had previously been given bicalutamide, and the other pre-docetaxel sequential treatments included flutamide (*n* = 74, 69%), enzalutamide (*n* = 38, 36%), estrogen (*n* = 34, 32%), and abiraterone (*n* = 20, 19%) in descending order. The median (IQR) serum GGT at docetaxel initiation was 31 (19–51) U/L.

### 3.2. Pre-Therapeutic Serum GGT and Other Clinicopathological Parameters for Predicting OS

**Figure 1:**
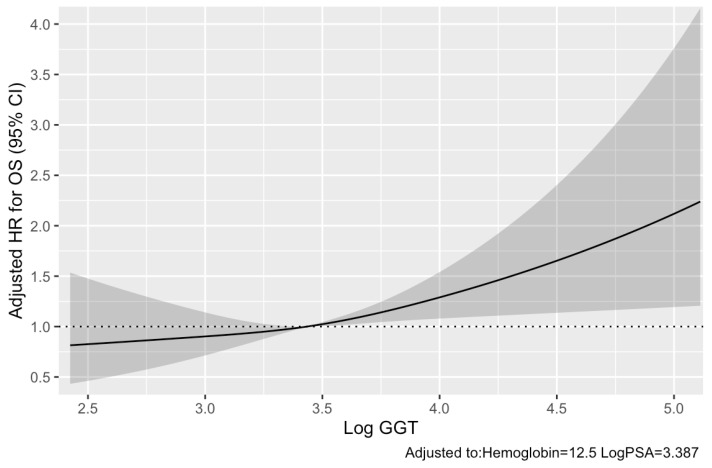
Relationship between Log GGT and adjusted HR for OS as visualized by splines with three knots located at the 25th, 50th, and 75th percentiles (adjusted to hemoglobin and Log PSA). CI, confidence interval; GGT, γ-glutamyltransferase; HR, hazard ratio; OS, overall survival; PSA, prostate-specific antigen.

During the median (IQR) follow-up period of 22.5 (12.3–50.6) months for survivors, 67 (63%) died. Associations of each variable with OS are listed in Table 2. Multivariable analysis demonstrated that the following variables were independently associated with adverse OS: low hemoglobin (hazard ratio [HR] 0.79, *p* = 0.002), high log GGT (HR 1.49, *p* = 0.006), and high Log PSA (HR 1.40, *p* < 0.001). Moreover, the relationship between OS and Log GGT, adjusted to hemoglobin and Log PSA, was visually examined using splines (Figure 1). Furthermore, C-indices of the updated Halabi and Smaletz models increased from 0.772 to 0.787 (*p* = 0.066) and from 0.777 to 0.785 (*p* = 0.118) by adding serum GGT to these models, respectively.

### 3.3. Pre-Therapeutic Serum GGT and Other Clinicopathological Parameters for Predicting PSA Response in 78 mCRPC Men Treated with Four or More Cycles of Docetaxel (Subcohort)

A total of 78 men with mCRPC received four or more cycles of docetaxel and their demographics are listed in Table 1. The median (IQR) PSA changes at 12 weeks after docetaxel initiation was −23.4% (−74.5% to 56.2%), and 33 (42%) men showed PSA response. Unlike OS, high Log GGT was not significantly associated with worse PSA response (odds ratio 1.50, *p* = 0.188; Table 3), while none of other factors was significantly associated with worse PSA response at 12 weeks after docetaxel initiation.

### 3.4. Pre-Therapeutic Serum GGT and Other Clinicopathological Parameters for Predicting PFS

A total of 100 (93%) men showed PSA or radiographical progression. Of these, radiographical progression preceded PSA progression in 6 (6%) men. Unlike OS, high Log GGT was not significantly associated with adverse PFS (univariable HR 1.06, *p* = 0.597; Table 4). Low albumin (HR 0.58, *p* = 0.037), and high Log PSA (HR 1.26, *p* < 0.001) were independently associated with adverse PFS.

### 3.5. Tissue GGT Expression Status and Clinicopathological Characteristics including Serum GGT Levels in 29 De Novo mCSPC Men

**Figure 2:**
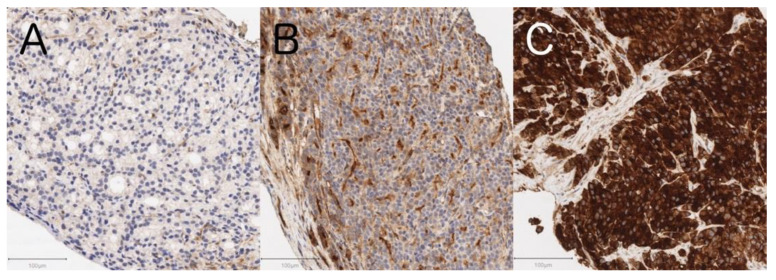
Representative histologic micrographs of immunohistochemical staining for GGT in prostate cancer cells showing (**A**) negative to weak (n = 6; 21%), (**B**) moderate (n = 12; 41%), and (**C**) strong (n = 11; 38%) GGT expression. Scale bars: 100 μm.

Among the prostate specimens from 29 de novo mCSPC men, 6 (21%), 12 (41%), and 11 (38%) showed negative to weak (Figure 2A), moderate (Figure 2B), and strong (Figure 2C) GGT expression, respectively. There was good inter-observer concordance for the independent evaluation of immunohistochemical staining by two authors (M.U. and K.T.) as verified by a Cohen’s kappa coefficient of 0.73 (95% CI 0.52–0.95). Of note, tissue GGT expression status significantly and moderately positively correlated with serum GGT levels (Spearman ρ = 0.53, *p* = 0.003). Gleason score or metastatic sites were not associated with tissue GGT expression status (Table 5).

## 4. Discussion

High serum GGT was significantly and independently associated with adverse OS in 107 men with mCRPC receiving docetaxel therapy. In addition, incorporation of serum GGT into established prognostic models (i.e., Halabi and Smaletz models) improved their accuracy for predicting OS in terms of C-indices, albeit not in a statistically significant manner (*p* = 0.066 and *p* = 0.118, respectively). These findings might support the notion that high serum GGT would potentially become a prognostic biomarker in men with mCRPC receiving docetaxel therapy. Similar results were previously reported in men with mCRPC receiving enzalutamide therapy [29]. In that study cohort, high serum GGT was independently associated with adverse OS regardless of sequential docetaxel therapy. Taken together, serum GGT could become a universal prognostic biomarker in men with mCRPC regardless of therapeutic regimens they receive.

Despite its significance in OS, pre-therapeutic serum GGT was not associated with PSA response or PFS in this study. PSA levels are known to reflect androgen receptor-driven proliferation in PC cells [30]. However, androgen blockade is not the docetaxel’s direct mechanism of action as docetaxel binds to the β-tubulin subunit of depolymerization, arrests the cell cycle during G2/M, and leads to cell death [31]. Therefore, PSA may not immediately reflect actual disease progression following docetaxel therapy, which can also be supported by the fact that a quarter of men with mCRPC had radiographic progression without PSA failure [32]. Moreover, PSA flare phenomenon has been reported in patients with mCRPC receiving docetaxel therapy with lack of clinical progression [33]. Furthermore, the association between PFS and OS was especially weak in the early follow-up period, and early changes in PSA or imaging may not reflect true failure of docetaxel therapy [34]. On the contrary, our previous study on men with mCRPC receiving enzalutamide therapy demonstrated that high serum GGT predicted not only an adverse prognosis but also poorer PSA response, maximal PSA change, and PSA-PFS [29]. Even though radiographic progression was included in the definition of PFS in the current analysis, radiographic examinations were not provided to the present patient cohort at periodic intervals unlike PSA measurement. Therefore, more frequent radiographic examinations may have yielded different results in terms of therapeutic response to docetaxel.

Our hypothesis that serum GGT might be associated with response to docetaxel was generated based on interesting preclinical findings that GGT-positive PC3 cells were more resistant to the toxicity of cisplatin than GGT-negative PC3 cells, since the former could cleave extracellular GSH and thus provided additional cysteine required for diminishing the tumor toxicity of cisplatin [21]. Nonetheless, serum GGT was not associated with PSA response or PFS after docetaxel initiation in our study. One of possible explanations is that PC cells would grow more slowly in clinical settings than in experimental settings, where expression of GGT benefited tumors which grew so rapidly that access to cysteine was limiting for growth. Another reason would lie in differences in primary mechanisms of action of cisplatin and docetaxel. The degree of resistance to cisplatin, reacting with the cellular deoxyribonucleic acid by binding to nucleotides, has been reported to be correlated with intracellular GSH levels as maintained by enzymatic activities of GGT [35]. Moreover, a prodrug named eprenetapopt, also known as APR-246, enhanced the apoptotic response to cisplatin by decreasing intracellular GSH levels, whereas no synergetic effects were observed in combination with docetaxel [36]. Therefore, GSH-independent apoptosis pathways which are independent of GGT activities may contribute to the mechanisms of action of docetaxel. Further basic and clinical research is required to clarify pharmacological roles of GGT in men with mCRPC receiving docetaxel therapy.

Serum GGT could be a specific biomarker reflecting the tumor GGT expression, given that most PC specimens (79%) showed moderate to strong GGT expression in the immunohistochemical analysis (Table 5) and that serum GGT could increase in patients with advanced cancer with GGT overexpression according to a recent systematic review [15]. GGT is an ectoenzyme known to contribute to the metabolism of GSH, playing a critical physiological role in protecting cells against oxidative stress [11]. GGT is reportedly expressed in the cell surface of normal prostatic glands and salvages constituent amino acids of GSH from ductal fluids [16,37]. The oxidative stress mediated by GGT has been suggested to underlie etiological mechanisms leading to adverse disease outcomes [11,38]. This study demonstrated a positive correlation between GGT expression status in PC tissues and serum GGT. Although the source of serum GGT has not been fully understood, cultured human PC cells reportedly release a specific fraction of GGT (i.e., big-GGT with the molecular weight of 2000 kDa), whose activity increases in parallel with cell growth [17]. GGT expression in PC tissues may be more strongly associated with disease aggressiveness than serum GGT, given that tissue GGT expression status can more directly reflect the phenotype of cancer cells. There are several selective GGT inhibitors including acivicin, azaserine, and 6-diazo-5-oxonorleucine [39]. Thus, men with PC with high GGT expression could become good candidates for future targeted therapy utilizing these inhibitors. Because currently available GGT inhibitors are highly toxic, further clinical studies on less toxic GGT inhibitors, such as OU749 and ovothiols, are desirable to develop a potential GGT-targeted therapy in the future [40,41]. Notwithstanding, vigorous prospective and experimental studies are warranted before clinical application of these agents.

In the present survival analysis, there were other prognostic biomarkers than high GGT in men with mCRPC receiving docetaxel therapy, including low hemoglobin and high PSA, both of which had been reported to be significant prognosticators in previous studies [7,8,9]. Of note, opioid use, low albumin, high ALP, and high LDH were significantly associated with adverse OS in univariable analysis but not in multivariable analysis. Independent prospective cohorts of men with mCRPC receiving docetaxel therapy are required before implementing our findings into clinical practice according to the REporting recommendations for tumor MARKer prognostic studies (REMARK) guideline [42].

Several limitations exist in the present study. Firstly, our findings are preliminary since they are from a retrospective, single-institutional study with a relatively small patient cohort consisting of only Japanese men. Validation of patient data from various countries is needed in order to assess the generalizability of our findings. Secondly, treatments given before docetaxel therapy greatly varied in our cohort (e.g., the number of pre-docetaxel sequential treatments). Nonetheless, our cohort would reflect the real-world population whose backgrounds are more heterogenous than those in clinical trials. Thirdly, we evaluated PSA response in 78 men receiving four or more cycles of docetaxel. Admitting that this subcohort may be biased toward a healthier population, we only included them so that their PSA measurements were available at 12 weeks (i.e., four cycles) after docetaxel initiation as defined by the Prostate Cancer Clinical Trials Working Group 3 [26]. Fourthly, the status of serum GGT kinetics during docetaxel therapy was not considered in the present study although serum GGT kinetics could be informative in predicting survival as it could evaluate the tumor burden successively [43]. Thus, kinetic analysis of serum GGT in men with mCRPC during docetaxel therapy is worth conducting in the future. Fifthly, we only investigated expression status of GGT1 in this study even though there are seven other potential full-length GGT protein family members, of which only GGT1 and GGT5 have thus far been proven to be catalytically active [44]. Therefore, further immunohistochemical analysis for other related enzymes including GGT5 may yield different results. Despite these limitations, the present study demonstrated that serum GGT could be a prognostic biomarker in men with mCRPC receiving docetaxel therapy, and that high serum GGT would be derived from overexpressed GGT in PC tissues. 

## 5. Conclusions

The present study for the first time demonstrated that serum GGT could be a prognostic biomarker in men with mCRPC receiving docetaxel therapy, and that overexpressed GGT in PC cells might be responsible for the elevation of serum GGT. Not only clinical applications of GGT for more accurate prognostication in men with mCRPC receiving docetaxel therap but also development of GGT-targeted therapy would be desirable in the future.

## Figures and Tables

**Table 1:** Baseline characteristics of mCRPC men (subcohort: mCRPC men treated with four or more cycles of docetaxel).

Characteristic at Docetaxel Initiation	Full Cohort		Subcohort	
	(*n* = 107)		(*n* = 78)	
Age [years] *	72.6	(68.7–76.7)	71.2	(68.7–75.5)
ECOG PS				
0	84	(79%)	60	(77%)
≥1	23	(21%)	18	(23%)
Gleason sum				
<8	8	(7%)	7	(9%)
≥8	89	(83%)	64	(82%)
unknown	10	(9%)	7	(9%)
Metastatic sites				
Lymph node only (M1a)	7	(7%)	6	(8%)
Bone/bone + lymph node (M1b)	82	(77%)	58	(74%)
Any visceral (M1c)	18	(17%)	14	(18%)
Opioid use				
Yes	19	(18%)	13	(17%)
No	88	(82%)	65	(83%)
Pre-docetaxel sequential treatments				
Bicalutamide	107	(100%)	78	(100%)
Flutamide	74	(69%)	58	(74%)
Estrogen	34	(32%)	24	(31%)
Enzalutamide	38	(36%)	21	(27%)
Abiraterone acetate	20	(19%)	12	(15%)
Number of pre-docetaxel treatments *	2	(1–3)	1	(1–2)
Time from diagnosis to docetaxel [years] *	2.5	(1.1–4.4)	2.4	(1.0–4.0)
Laboratory parameters				
Hemoglobin [g/L] *	125	(113–133)	126	(114–133)
Albumin [g/L] *	41	(38–44)	41	(38–44)
GGT [U/L] *	31	(19–51)	31	(19–50)
LDH [U/L] *	201	(179–253)	198	(179–254)
ALP [U/L] *	393	(252–618)	356	(249–543)
CRP [mg/L] *	2.7	(1.0–7.0)	2.6	(1.0–7.0)
PSA [ng/mL] *	29.6	(7.3–121.2)	19.3	(4.6–93.5)
Cycles of docetaxel				
<4	29	(27%)	0	(0%)
≥4	78	(73%)	78	(100%)
Post-docetaxel sequential treatments				
Flutamide	1	(1%)	0	(0%)
Estrogen	34	(32%)	30	(38%)
Enzalutamide	38	(36%)	31	(40%)
Abiraterone acetate	30	(28%)	21	(27%)
Cabazitaxel	22	(21%)	16	(21%)
Radium-223	5	(5%)	4	(5%)

ALP, alkaline phosphatase; CRP, C-reactive protein; ECOG PS, Eastern Cooperative Oncology Group performance status; GGT, γ-glutamyltransferase; IQR, interquartile range; LDH, lactate dehydrogenase; mCRPC, metastatic castration-resistant prostate cancer; PSA, prostate-specific antigen. * Median (IQR).

**Table 2:** Univariable and multivariable analyses for OS in 107 mCRPC men treated with docetaxel.

Factor	Univariable			Multivariable (Final Model)		
	HR	(95% CI)	*p*	HR	(95% CI)	*p*
Age	1.01	(0.97–1.05)	0.566			
ECOG PS (≥1 vs. 0)	1.62	(0.92–2.85)	0.092			
Gleason sum (≥8 vs. <8)	0.98	(0.50–1.93)	0.957			
Visceral metastasis (Yes vs. no)	0.87	(0.45–1.66)	0.664			
Opioid use (Yes vs. no)	2.57	(1.43–4.62)	0.002			
Number of pre-docetaxel treatments	1.11	(0.85–1.44)	0.439			
Time from diagnosis to docetaxel	0.98	(0.91–1.06)	0.662			
Hemoglobin	0.72	(0.63–0.82)	<0.001	0.79	(0.68–0.92)	0.002
Albumin	0.30	(0.15–0.61)	<0.001			
Log GGT	1.37	(1.06–1.78)	0.017	1.49	(1.12–1.98)	0.006
LDH (>ULN vs. ≤ULN)	2.35	(1.38–4.00)	0.002			
Log ALP	1.90	(1.39–2.60)	<0.001			
CRP	1.11	(0.99–1.24)	0.067			
Log PSA	1.52	(1.32–1.76)	<0.001	1.40	(1.21–1.62)	<0.001

ALP, alkaline phosphatase; CI, confidence interval; CRP, C-reactive protein; ECOG PS, Eastern Cooperative Oncology Group performance status; GGT, γ-glutamyltransferase; HR, hazard ratio; LDH, lactate dehydrogenase; PSA, prostate-specific antigen; ULN, upper limit of normal.

**Table 3:** Univariable analysis for PSA response at 12 weeks after docetaxel initiation in 78 mCRPC men treated with four or more cycles of docetaxel.

Factor	OR	(95% CI)	*p*
Age	1.01	(0.94–1.08)	0.767
ECOG PS (≥1 vs. 0)	1.21	(0.41–3.57)	0.731
Gleason sum (≥8 vs. <8)	4.94	(0.56–43.50)	0.150
Visceral metastasis (Yes vs. no)	0.46	(0.13–1.61)	0.222
Opioid use (Yes vs. no)	1.66	(0.50–5.51)	0.408
Number of pre-docetaxel treatments	0.64	(0.39–1.05)	0.075
Time from diagnosis to docetaxel	0.99	(0.86–1.14)	0.855
Hemoglobin	1.13	(0.84–1.52)	0.421
Albumin	2.69	(0.81–8.94)	0.105
Log GGT	1.50	(0.82–2.74)	0.188
LDH (>UNL vs. ≤ULN)	1.09	(0.39–3.05)	0.868
Log ALP	1.05	(0.60–1.85)	0.865
CRP	0.95	(0.74–1.21)	0.671
Log PSA	0.89	(0.71–1.11)	0.298

ALP, alkaline phosphatase; CI, confidence interval; CRP, C-reactive protein; ECOG PS, Eastern Cooperative Oncology Group performance status; GGT, γ-glutamyltransferase; LDH, lactate dehydrogenase; OR, odds ratio; PSA, prostate-specific antigen; ULN, upper limit of normal.

**Table 4:** Univariable and multivariable analyses for PSA and radiological PFS in 107 mCRPC men treated with docetaxel.

Factor	Univariable			Multivariable		
	HR	(95% CI)	*p*	HR	(95% CI)	*p*
Age	1.02	(0.99–1.05)	0.121			
ECOG PS (≥1 vs. 0)	0.90	(0.56–1.45)	0.672			
Gleason sum (≥8 vs. <8)	0.62	(0.37–1.05)	0.073			
Visceral metastasis (Yes vs. no)	0.88	(0.51–1.53)	0.656			
Opioid use (Yes vs. no)	1.38	(0.83–2.30)	0.210			
Number of pre-docetaxel treatments	1.31	(1.09–1.57)	0.005			
Time from diagnosis to docetaxel	1.02	(0.97–1.08)	0.498			
Hemoglobin	0.83	(0.74–0.92)	<0.001			
Albumin	0.39	(0.23–0.66)	<0.001	0.58	(0.35–0.97)	0.037
Log GGT	1.06	(0.85–1.34)	0.597			
LDH (> UNL vs. ≤ ULN)	1.79	(1.16–2.79)	0.009			
Log ALP	1.53	(1.21–1.93)	< 0.001			
CRP	1.11	(1.00–1.22)	0.045			
Log PSA	1.30	(1.18–1.43)	< 0.001	1.26	(1.14–1.40)	< 0.001

ALP, alkaline phosphatase; CI, confidence interval; CRP, C-reactive protein; ECOG PS, Eastern Cooperative Oncology Group performance status; GGT, γ-glutamyltransferase; HR, hazard ratio; LDH, lactate dehydrogenase; PSA, prostate-specific antigen; ULN, upper limit of normal.

**Table 5:** Clinicopathological characteristics of 29 de novo mCSPC men according to GGT expression status.

Characteristic at Diagnosis	Tissue GGT Expression Status						*p*
	Negative to Weak (*n* = 6)		Moderate (*n* = 12)		Strong (*n* = 11)		
Age [years], median (IQR)	76.2	(65.2–79.6)	69.6	(67.0–72.4)	70.5	(64.6–78.2)	0.438 *
Gleason score, median (IQR)	9	(9–9)	9	(9–9)	9	(9–9)	0.772 *
Metastatic sites							0.492 ^†^
Lymph node only (M1a)	0	(0%)	0	(0%)	0	(0%)	
Bone/bone + lymph node (M1b)	5	(83%)	9	(82%)	10	(100%)	
Any visceral (M1c)	1	(17%)	2	(18%)	0	(0%)	
Serum GGT [U/L], median (IQR)	15	(10.5–33.3)	33	(23.5–53.3)	64	(50–74)	0.018 *

GGT, γ-glutamyltransferase; IQR, interquartile range. * Kruskal–Wallis test. ^†^ Fisher’s exact test.

## Data Availability

Data sharing is not applicable to this article.
